# Organizational resilience and primary care nurses’ work conditions and well-being: a multilevel empirical study in China

**DOI:** 10.1093/heapol/czae091

**Published:** 2024-09-16

**Authors:** Wenhua Wang, Mengyao Li, Jinnan Zhang, Ruixue Zhao, Huiyun Yang, Rebecca Mitchell

**Affiliations:** School of Public Policy and Administration, Xi’an Jiaotong University, No.28 West Xianning Road, Xi’an 710049, China; School of Public Policy and Administration, Xi’an Jiaotong University, No.28 West Xianning Road, Xi’an 710049, China; School of Public Policy and Administration, Xi’an Jiaotong University, No.28 West Xianning Road, Xi’an 710049, China; School of Public Policy and Administration, Xi’an Jiaotong University, No.28 West Xianning Road, Xi’an 710049, China; School of Public Policy and Administration, Xi’an Jiaotong University, No.28 West Xianning Road, Xi’an 710049, China; Health and Wellbeing Research Unit (HoWRU), Macquarie Business School, Macquarie University, 3 Management Drive, Sydney 2109, Australia

**Keywords:** Organizational resilience, adaptive capacity, planning capacity, working conditions, well-being, primary care, nurses

## Abstract

Resilience is crucial for a health system to better prevent and respond to public health threats and provide high-quality services. Despite the growing interest in the concept of resilience in health care, however, there is little empirical evidence of the impact of organizational resilience, especially in primary care settings. As the largest professional group in primary care, primary care nurses are taking more and more responsibilities during their daily practice, which influences both their work conditions and well-being. This study aims to examine the association between organizational resilience and primary care nurses’ working conditions and well-being. Using a convenience sampling approach, we recruited 175 primary care nurses from 38 community health centres (CHCs) in four cities in China. Organizational resilience was operationalized as comprising two domains: adaptive capacity and planning capacity, and measured using a 16-item scale. The primary care nurses’ working condition indicators comprised variables of psychological safety, organizational commitment, professional commitment, and self-directed learning; well-being indicators included depression and burn-out. Hierarchical linear regression models were built for analysis. We found that the sampled CHCs have a relatively high level of organizational resilience. The organizational resilience was positively associated with the four indicators of working conditions: psychological safety (*β* = 0.04, *P* < 0.01), organizational commitment (*β* = 0.38, *P* < 0.01), professional commitment (*β* = 0.39, *P* < 0.01), and self-directed learning (*β* = 0.28, *P* < 0.01). However, organizational resilience was not significantly associated with the two well-being indicators. Furthermore, we found that the adaptive capacity has stronger association compared with planning capacity. Therefore, primary care manager should build resilient organizations, especially the adaptive capacity, in order to enhance primary care nurses’ psychological safety, commitment and learning behaviours. Further studies should also be conducted to understand the link between organizational resilience and primary care nurses’ well-being.

Key messages

- Resilient health system building is important to address the global health challenges. Little is known about the impact of organizational resilience in primary care, especially in developing countries including China.
- As the largest professional group in primary care, primary care nurses are taking more and more responsibilities during their daily practice, which influences both their work conditions and well-being.
- Organizational resilience was positively associated with primary care nurses’ work conditions, including psychological safety, organizational commitment and professional commitment and self-directed learning. However, organizational resilience was not associated with primary care nurses’ well-being, including depression and work exhaustion.
- For the two domains of organizational resilience, adaptive capacity has a stronger influence compared with planning capacity.

## Introduction

Resilience is generally taken to mean a system’s ability to continue to meet its objectives in the face of challenges, emphasizing a system’s capacity not just to withstand shocks but also to adapt and transform (Barasa *et al*., 2018). Recently, the concept of resilience has gained new momentum in organization studies (Hillmann and Guenther, 2021). Organizational resilience has been defined as ‘the maintenance of positive adjustment under challenging conditions such that the organization emerges from those conditions strengthened and more resourceful’ (Barasa *et al*., 2018). Organizations can demonstrate resilience in the face of challenges in many ways, from reconfiguring resources and reshaping relationships to optimizing processes and changing protocols and guidelines (Barasa *et al*., 2018; Ignatowicz *et al*., 2023). Research with business and management disciplines suggests that organizational resilience links to reduced organizational failure and decline, increased efficacy, organizational performance and competitive advantage (Hillmann and Guenther, 2021).

As the global healthcare systems are facing significant chronic challenges such as ageing populations, multimorbidity, limited human resource, constrained finances, as well as acute shocks such as the Coronavirus Disease 2019 (COVID-19) pandemic (Gilson *et al*., 2017; Barasa *et al*., 2018), policymakers, healthcare organizations and health professionals advocate expanding the scope of practice of the nurse workforce to better address these challenges (Norful *et al*., 2017; Poghosyan *et al*., 2017; Rice *et al*., 2017; Coster *et al*., 2018; Laurant *et al*., 2018). These reforms lead to more responsibility and new work content for primary care nurses, which influence both their work conditions and well-being, key predictors of high-quality service provision. For example, during the pandemic, primary care nurses not only provided regular care but also took increasing responsibility for pandemic-related care, such as vaccination rollout, community monitoring and testing and health education (Norful *et al*., 2017; Ashley *et al*., 2022). These changes led to considerable uncertainty, high infection risk and heavy workload, which ultimately diminished their work condition and well-being (Al Maqbali *et al*., 2021). A recent study reported that a considerable proportion of primary care nurses experienced anxiety, depression or stress, with a pooled prevalence rate of 39.6% during the pandemic (Halcomb *et al*., 2022).

Resilient health care is an emerging area of theory and research to understand how healthcare organizations cope with the dynamic, variable and demanding environments in which they operate to maintain high-quality care based on insights from complexity and systems theory (Janet *et al*., 2020). Building resilient primary care organizations is likely to represent a feasible approach to protecting primary care nurses’ well-being and work conditions and maintaining the capacity and performance of their regular service provision, especially during the pandemic. A resilient organization has strong planned capacity to reserve enough resources and build an efficient network and can provide strong support to their health professionals to address the challenges. Meanwhile, they also have strong adaptive capacity to reconfigure resources and reshape relationships and workflow to ensure that their health professionals know what to do and how to work. These features will create a psychologically safe environment and a dynamic learning climate, enhancing the connections between their health professionals with the organizations and improving well-being (Barasa *et al*., 2018; Domínguez-Salas *et al*., 2021; Ignatowicz *et al*., 2023).

Therefore, we hypothesized that organizational resilience is positively associated with work conditions and well-being among primary care nurses. This study aims to examine the association between organizational resilience and primary care nurses’ working conditions and well-being. The results contribute to existing research by revealing potential organizational and practice interventions to improve primary care nurses’ work performance and well-being.

## Methods

### Data sources

This cross-sectional study was conducted in four large cities in China: Shanghai, Tianjin, Shenzhen and Jinan. Located in the east of China, these cities have relatively well-developed primary care systems with high-quality infrastructure and qualified primary care professionals. The results from this study could inform further studies with designs for more generalizable sampling frames in China and other countries.

Using the convenience sampling approach, the selection of community health centres (CHCs) in each city was suggested by the local health bureau staff based on the practice size and service performance levels of CHCs in their respective catchment areas. The sample size was determined based on the requirements of multilevel regression models and the feasibility of data collection during the pandemic. Finally, 38 CHCs in these four cities were selected: 10 in Shanghai, 14 in Shenzhen, 8 in Tianjin and 6 in Jinan.

This survey was conducted from November 2021 to May 2022. With the coordination of local health bureau staff, in each CHC, all primary care nurses on duty the day the researchers arrived were invited to complete an individual survey, which included information on their personal characteristics, working conditions and well-being. The CHC directors were invited to complete an organizational survey, including information on organizational characteristics, such as human resources, ownership, medical equipment and organizational resilience.

The individual survey and organizational survey were conducted in different meeting rooms. Research assistants distributed the self-administered paper questionnaires to the participants after explaining the purpose of this study and the content of this survey. After the participants completed the questionnaire, they returned it to the research assistants directly in order to reduce the risk of external influence on responses. The research assistants would check if there was any missing information to ensure the completeness of each questionnaire. All 175 primary care nurses present at the 38 CHCs completed the individual survey, and the 38 CHC directors completed the CHC organizational survey.

### Measures

#### Organizational resilience

In line with previous studies on organizational resilience, we operationalized organizational resilience as comprising two factors: adaptive capacity and planning capacity (Mcmanus *et al*., 2008; Lee *et al*., 2013). Based on this concept, we measured adaptive and planning capacity through eight items, respectively, to measure organizational resilience (Wang and Cai, 2019). Responses were provided on a five-Likert scale, with anchors from 1 (strongly disagree) to 5 (strongly agree). The average score of 16 items was the score on organizational resilience, with higher scores indicating enhanced organizational resilience.

The primary care nurses’ working condition indicators comprised four variables of psychological safety, organizational commitment, professional commitment and self-directed learning; well-being indicators included depression and burn-out. The details of instruments measuring these indicators were reported in the Supplementary file.

#### Psychological safety

A previously validated 7-item instrument was used to measure psychological safety. Responses were provided on a 7-Likert scale, ranging from 1 (strongly disagree) to 7 (strongly agree) (Edmondson, 1999). The scores in the first three items were reversed before analysis. The average score of seven items was the score of psychological safety.

#### Organizational commitment

The affective organizational commitment was measured using the six-item scale from the study by Meyer et al., which was developed from nurses with good reliability (Meyer *et al*., 1993). Responses were averaged for individual scores of six items ranging from 1 (strongly disagree) to 5 (strongly agree).

#### Professional commitment

To measure the professional commitment of the registered nurses, two items from Blau’s scale were used (Blau, 1988). Each item was reported with anchors ranging from 1 (strongly disagree) to 5 (strongly agree). The average score of the two-item scores represented the professional commitment level of primary care nurses.

#### Self-directed learning

Qiu et al’s scale with three items developed in Chinese context was used to measure self-directed learning (Qiu *et al*., 2016). Each item was reported on a five-Likert scale ranging from 1 (strongly disagree) to 5 (strongly agree). The average score of the three-item scores represented the level of self-directed learning.

#### Depression

The Patient-Reported Outcomes Measurement Information System (PROMIS) depression four-item short form was widely used to measure the depression of adults (Pilkonis *et al*., 2011). The response to each answer ranged from 1 (not at all) to 5 (completely), and the raw score was the sum of scores within four items ranging from 4 to 20. In order to compare with other PROMIS domains, we translated the raw score to the *T*-score which was the standardized score, and the *T*-score ranged from 41 to 79.

#### Burn-out

Burn-out was measured using the instrument of the professional fulfilment index (Trockel *et al*., 2018). Responses ranged from 1 (strongly disagree) to 5 (strongly agree), and the average score of four items was the score in burn-out of nurses.

#### Covariates

There were two types of covariates: personal characteristics and organizational characteristics. The personal characteristics included nurses’ age, gender, marital status (married or cohabiting, divorced or widowed), education level and tenured position. The organizational characteristics included organizational ownership (government-managed or public hospital–managed), organizational size (number of CHC staff), number of medical equipment valued over $1600 and the percentage of health professionals.

#### Statistical analysis

First, descriptive analysis was performed to investigate personal and organizational characteristics. Frequency and percentage for categorical variables and mean and standard deviation for continuous variables were used. Second, analyses for organizational resilience, work condition and well-being indicators were conducted, including description with mean and standard deviation, reliability with Cronbach coefficient and the Pearson correlation coefficients among these variables. Next, because primary care nurses nested in CHCs, we used the hierarchical linear regression model (HLM) to test the relationship between organizational resilience and work condition and well-being indicators. All the continuous variables were centred at the grand mean to reduce collinearity and facilitate interpretation (Enders and Tofighi, 2007). Before HLM, we tested collinearity by calculating the variance inflation factor (VIF) value and tested whether HLM could be adapted by calculating the intraclass correlation coefficient (ICC). The ICC value reflected the extent to which the variation in an outcome variable could be attributed to the higher CHC-level factors, with over 0.059 indicating that HLM was appropriate for the analysis (Cohen, 1988). Among 175 primary care nurses, one sample was dropped because of missing data on marriage status and education level. In the remaining 174 samples, there were some missing data in measuring self-directed learning, and we used mean value for imputation of the missing data (Austin *et al*., 2021). The *P* value < 0.05 was considered statistically significant. All analyses were performed using Stata 15.1.

## Results

### Characteristics of the participants

Responses from 174 primary care nurses from 38 CHCs were included in final analysis. The characteristics of primary care nurses and their serving CHCs are detailed in Table 1. The average age of participant nurses was 34 years, over 98% was female and almost 15% of nurses were unmarried. Regarding the education level, over 90% of nurses received a college education or above. Nearly half of the nurses held a tenured position. Over 60% of participants were working in government-managed CHCs. Nearly 30% was working in CHCs with over 100 staff. Over half of participants worked in CHCs with no more than 10 pieces of equipment valued over $1600.

**Table 1.** Description of nurses and organizational characteristics

Characteristics	*n* (%)/mean (SD)
Nurse characteristics	
Age, years, mean (SD)	33.98 (7.79)
Gender, *n* (%)	
Male	3 (1.72)
Female	171 (98.28)
Marriage, *n* (%)	
No	27 (14.3)
Yes	149 (85.63)
Education level, *n* (%)	
High school or below	11 (6.32)
College or above	163 (93.68)
Tenured position, *n* (%)	
No	95 (54.60)
Yes	79 (45.40)
Organization characteristics	
Organizational ownership, *n* (%)	
Government	109 (62.64)
Public hospital	65 (37.36%)
Percentage of health professionals, mean (SD)	88 (0.09)
Organizational size, *n* (%)	
≤35	47 (27.01%)
36–55	32 (18.39%)
56–100	44 (25.29%)
>100	51 (29.31)
No. of valuable medical equipment, *n* (%)	
≤4	35 (20.11)
5–10	59 (33.91)
11–30	23 (13.22)
>30	57 (32.76)

### Correlation and reliability of the study variables

Correlation coefficients and the Cronbach *α* coefficients of the study variables are shown in Table 2. The mean score of the variables was 4.29 out of 5 for organizational resilience, 5.22 out of 7 for psychological safety, 4.05 out of 5 for organizational commitment, 3.70 out of 5 for professional commitment, 4.17 out of 5 for self-directed learning, 52.49 out of 79 for depression and 2.39 out of 5 for burn-out. All these study variables exhibited good reliability with Cronbach *α* coefficients over 0.8, except for professional commitment with 0.76. Organizational resilience had a significant correlation with organizational commitment, professional commitment and self-directed learning.

**Table 2.** Mean (*M*), SD, correlations and reliabilities of the variables

Variable	Items	*M*	SD	OR	PS	OC	PC	SL	D	B
Organizational resilience	16	4.29	0.61	0.93						
Psychological safety	7	5.22	1.11	0.108	0.83					
Organizational commitment	5	4.05	0.86	0.185*	0.464^**^	0.94				
Professional commitment	5	3.70	1.01	0.158*	0.411^**^	0.673^**^	0.76			
Self-directed learning	5	4.17	0.71	0.155*	0.431^**^	0.463^**^	0.425^**^	0.82		
Depression	4	52.49	8.41	−0.11	−0.517^**^	−0.415^**^	−0.428^**^	−0.356^**^	0.90	
Burn-out	5	2.39	1.00	−0.035	−0.417^**^	−0.441^**^	−0.447^**^	−0.302^**^	0.544^**^	0.87

Reliability coefficients, Cronbach alpha, appear along the diagonal.

Abbreviations: B, Burn-out; D, depression; OC, organizational commitment; OR, organizational resilience; PC, professional commitment; PS, psychological safety; SL, self-directed Learning.

* *P* < 0.05.;

** *P* < 0.001.

### The role of organizational resilience among primary care nurses

Before building HLMs, we performed collinearity testing, finding that all VIF values were <5, evidencing support that our data were not biased due to multicollinearity. The ICC values in all hierarchical null models were all over 0.059, indicating that hierarchical linear regression modelling was appropriate in this study. The results of HLMs are given in Table 3.

**Table 3.** Hierarchical linear regression models examining the association between organizational resilience and primary care nurse indicators

	Psychological safety		Organizational commitment		Professional commitment		Self-directed Learning		Depression		Burn-out	
Key organizational variable	*β*	SE	*β*	SE	*β*	SE	*β*	SE	*β*	SE	*β*	SE
Organizational resilience	0.40**	0.15	0.38**	0.12	0.39**	0.13	0.28**	0.10	−1.50	1.24	−0.09	0.15
Control variables												
Personal level												
Age	0.00	0.01	0.02*	0.01	0.02*	0.01	0.01	0.01	−0.01	0.10	−0.02	0.01
Gender (ref = female)												
Male	−0.32	0.59	−0.20	0.47	0.13	0.56	−0.26	0.40	3.13	4.86	−0.20	0.56
Marriage (ref = no)												
Yes	−0.69**	0.23	0.09	0.19	−0.14	0.22	−0.24	0.16	1.49	1.92	0.35	0.22
Education (ref = high school or below)												
College or above	0.15	0.33	0.02	0.27	−0.36	0.32	0.17	0.23	3.92	2.76	0.43	0.32
Tenured position (ref = no)												
Yes	0.15	0.21	−0.11	0.17	−0.27	0.20	0.09	0.14	0.49	1.70	0.22	0.20
Control variables												
Organizational level												
Ownership (ref = government)												
Public hospital	−0.15	0.29	−0.41	0.24	−0.40	0.25	−0.15	0.18	−0.05	2.37	0.18	0.29
Percentage of health professionals	2.79**	1.02	1.28	0.84	2.29*	0.91	0.55	0.65	−6.19	8.43	−0.99	1.01
Organizational size (ref ≤35)												
36–55	0.54	0.28	0.37	0.24	0.27	0.25	0.36*	0.18	−3.64	2.34	−0.25	0.28
56–100	0.10	0.34	0.14	0.29	0.11	0.30	−0.18	0.22	−3.54	2.85	−0.19	0.35
>100	0.29	0.34	0.36	0.28	0.35	0.29	−0.03	0.21	−3.37	2.78	−0.49	0.34
No. of valuable medical equipment (ref.=≤4)												
5–10	0.13	0.25	0.08	0.21	−0.13	0.22	0.22	0.16	2.16	2.05	0.39	0.25
11–30	−0.01	0.32	−0.26	0.27	−0.28	0.29	−0.05	0.21	3.19	2.68	0.66*	0.32
>30	−0.81*	0.32	−0.72**	0.26	−0.89**	0.28	−0.06	0.20	5.14*	2.62	0.90**	0.32

* *P* < 0.05.

** *P* < 0.01.

The results demonstrated that organizational resilience had a positive association with psychological safety (*β *= 0.40, *P *< 0.01), organizational commitment (*β *= 0.38, *P *< 0.01), professional commitment (*β *= 0.39, *P *< 0.01) and self-directed learning (*β *= 0.28, *P *< 0.01), which supported the hypothesis that organizational resilience was positively associated with primary care nurses’ working conditions. However, the organizational resilience was not significantly associated with depression and burn-out, which did not support the hypothesis that organizational resilience was positively associated with primary care nurses’ well-being.

With regard to the covariates at the personal level, age had a positive relationship with organizational commitment (*β *= 0.02, *P *< 0.05) and professional commitment (*β *= 0.02, *P *< 0.05). Compared with unmarried nurses, married nurses showed a negative association with psychological safety (*β* = −0.69*, P *< 0.01). Out of the covariates at the organizational level, the percentage of health professionals in CHC were significantly associated with psychological safety (*β *= 2.79, *P *< 0.01) and professional commitment (*β *= 2.29, *P *< 0.05). In contrast to organizational size of <35 people, the CHC size of 36–55 people was significantly associated with nurses’ self-directed learning (*β *= 0.36, *P *< 0.05). Compared with CHCs with valuable equipment under four pieces, CHCs with pieces over 30 were negatively associated with psychological safety (*β* = −0.81, *P *< 0.05), organizational commitment (*β *= −0.72, *P *< 0.01) and professional commitment (*β *= −0.89, *P *< 0.05), while they had a positive relationship with depression (*β *= 5.14, *P *< 0.05) and burn-out (*β *= 0.90, *P *< 0.01).

Table 4 shows the association of two dimensions of organizational resilience with primary care nurses’ working conditions and well-being. Adaptive capacity showed a significant positive association with the four indicators of working condition: psychological safety (*β *= 0.40, *P *< 0.01), organizational commitment (*β *= 0.41, *P *< 0.01), professional commitment (*β *= 0.45, *P *< 0.01) and self-directed learning (*β *= 0.32, *P *< 0.01). Meanwhile, planning capacity also demonstrated a positive association with these four indicators. However, neither adaptive capacity nor planning capacity was significantly associated with the two well-being indicators.

**Table 4.** HLMs examining the association of two domains of organizational resilience with primary care nurse indicators

	Psychological safety		Organizational commitment		Professional commitment		Self-directed Learning		Depression		Burn-out	
	*Β*	SE	*β*	SE	*β*	SE	*β*	SE	*β*	SE	*β*	SE
Adaptive capacity	0.40**	0.15	0.41**	0.13	0.45**	0.14	0.32***	0.10	−1.50	1.27	−0.06	0.15
Planning capacity	0.33*	0.14	0.29*	0.11	0.29*	0.12	0.20*	0.09	−1.24	1.11	−0.10	0.13

The same control variables as in Table 3 were included in the models.

* *P* < 0.05.

** *P* < 0.01.

*** *P* < 0.001.

## Discussion

Using data of 174 primary care nurses from 38 CHCs in China, we examined the potential role of organizational resilience among primary care nurses and found that organizational resilience was positively associated with primary care nurses’ working conditions, including psychological safety, organizational commitment and professional commitment and self-directed learning. However, organizational resilience was not associated with primary care nurses’ well-being, including depression and burn-out.

As China is moving towards building high-quality primary healthcaresystem through developing interprofessional family doctor teams as well as experimenting with different payment models, primary care professionals are facing complex challenges. As the largest primary care professional group, building psychological safe environments is very important to support primary care nurses to meet and cope with these challenges. As an individual’s perception concerning the consequences of risk-taking (Edmondson and Lei, 2014), a previous study showed that an atmosphere of psychological safety reduces nurse work-related stress, emotional exhaustion, cognitive impairment and interpersonal risk effects at the workplace (Ma *et al*., 2021). Interventions aimed at creating a psychologically safe environment have also been proposed at the team level in primary care, including leader and leader inclusiveness, open culture, boundary spanner, chairing meetings and strong interpersonal relationships (Domínguez-Salas *et al*., 2021; Remtulla *et al*., 2021). Organizational-level factors were found to have been largely omitted from extant research, and resilience, in particular, has remained unexplored until now (Remtulla *et al*., 2021). This study provided evidence that organizational resilience could be a potential intervention levers to build psychological safety among primary care nurses.

Organizational commitment is defined as the degree of involvement of a worker with a particular organization. Affective commitment, which refers to the positive elements of organizational commitment in the nursing context, is the dimension of commitment that is most strongly associated with the intention to remain in an organization, as well as with work involvement (Orgambidez *et al*., 2019). Previous studies show that low levels of organizational commitment have been linked to negative work outcomes, such as missed nursing care, poor-quality nursing care, decreased organizational citizenship behaviour and high turnover (Baek *et al*., 2019; Hwang *et al*., 2022). This study confirmed the positive association between organizational resilience and organizational commitment among primary care nurses, which points to the value of building resilient primary care organizations in enhancing organizational commitment, which likely increases nursing care and decreases turnover.

Nursing is a profession that requires a high level of professional commitment. Nurses must demonstrate strong professional commitment in order to defend patients’ rights and to perform their job in the best way (Duran *et al*., 2021). The concept of professional commitment in nursing is the commitment to provide the best nursing care, the tendency to promote the profession and responsibility to the professional issues. Previous studies suggest that high professional commitment positively affects the professional performance and behaviours of nurses and has positive outcomes for patients (Momeni and Khatooni, 2023). Due to the COVID-19 pandemic, various physical and psychological negative situations experienced by nurses have been shown to decrease their professional commitment (Duran *et al*., 2021; Momeni and Khatooni, 2023). Existing literature suggested numerous factors affecting professional commitment, such as working conditions, work–family conflict, socio-demographic characteristics (age, gender, marital status, etc.) and organizational obstruction (Duran *et al*., 2021; Goldfarb *et al*., 2021). This study confirmed the positive association between organizational resilience and professional commitment among primary care nurses and proposes a new organizational-level avenue for interventions enhancing professional commitment.

Although organizational citizenship behaviour is not a new concept in the literature, there are few studies focusing on organizational citizenship behaviour in nursing (Cetin and Yayan, 2022). Self-directed learning, reflecting primary care nurses’ motivation and behaviour to learn by themselves in order to improve care quality, is an important component of organizational citizenship behaviour and also a key element of learning organizations. There is international call for developing learning organizations in health to address the complex challenges of global health reform efforts. There is widespread consensus that learning healthcare organization could facilitate improved patient experiences, per capita health cost and population health outcomes (Rattray *et al*., 2020). This study confirmed that organizational resilience was positively associated with self-direct learning behaviours among primary care nurses and proposed that organizational resilience might be a feasible approach to facilitate learning organizations building in health care.

However, we did not find the statistically significant association between organizational resilience and primary care nurses’ well-being, including depression and burn-out, given the high prevalence of depressive symptoms among nurses (Wang *et al*., 2021; Zhang *et al*., 2021; Shi *et al*., 2022). As a multilevel construct (i.e. individual, team, organization and system), resilience studies among nurses mostly focus on individual level and find that many nurses present low levels of resilience and resilience inversely correlates with depression and burn-out (Castillo-González *et al*., 2024). Further study should be conducted to understand the link of resilience at different levels with nurses’ well-being in primary care. Meanwhile, although organizational resilience was not found directly associated with nurses’ well-being, previous studies suggest that work conditions are potential predictors of well-being (Rabatin *et al*., 2016). Therefore, further study should be conducted to examine if the indirect link of organizational resilience and well-being exists among primary care nurses.

For the two dimensions of organizational resilience, we found the adaptive capacity, referring to reconfiguration of resources and reshaping relationships and workflow to ensure that their health professionals know what to do and how to work and have stronger association with primary care nurses’ work condition indicators compared with planning capacity, which is consistent with previous research that adaptive resilience is more influential since it is more sustainable and effective in the context of uncertainty about what the future could bring, especially in organization and systems where resources are scarce (Forsgren *et al*., 2022). This points out the importance of adaptive capacity in resilient organization building. A recent review showed that the resilience of organizations was influenced by the following factors: material resources, preparedness and planning, information management, collateral pathways and redundancy, governance processes, leadership practices, organizational culture, human capital, social networks and collaboration (Barasa *et al*., 2018). Further studies should be conducted to understand how to leverage these factors to building primary care organization’s adaptive capacity.

In terms of covariates, we found that CHCs with the more quantity of valuable medical equipment were negatively associated with work condition indicators (psychological safety, organizational commitment and professional commitment), while positively associated with well-being indicators (depression and work exhaustion). In general, CHCs with more quantity of valuable medical equipment have larger patient volumes, which could increase the workload and stress levels of their health workers. Previous research showed that the workload and work stress of nurses were negatively associated with organizational or professional commitment (Lu *et al*., 2007; Li *et al*., 2021) and positively associated with higher depression and burn-out (Chen *et al*., 2020; Li *et al*., 2021). Therefore, primary care facilities’ patient volume and medical equipment capacity should be considered when designing the organizational resilience intervention programmes in primary care.

The following limitations should also be considered in interpreting our findings. First, due to the challenges of collecting data during the COVID-19 pandemic, we only recruited 174 primary care nurses from 38 CHCs in four cities with a cross-sectional survey although the sample size satisfied the requirement of statistical models. The results are applicable across these sampling sites and cannot necessarily be generalized to other primary care settings. Second, this is a cross-sectional study, where the results can only confirm the association between organizational resilience and primary care nurse outcome indicators instead of demonstrating causation. Third, a cross-sectional design offers no protection against the effects of confounding variables not explicitly included in the models, e.g. ‘the salary of the nurse’ is a variable that was not available and not controlled in the model. Furthermore, this is a quantitative study instead of a mixed method study, and therefore, it is difficult to understand why organizational resilience is positively associated with primary care nurses’ work conditions while not associated with their well-being indicators. Further qualitative studies might be conducted to understand the mechanism. Despite the several limitations to this study that we noted, this study provides empirical evidence about the importance of organizational resilience among primary care nurses using validated survey instruments and proposes a potential organizational lever to improve work conditions of primary care nurses.

## Conclusion

The sampled CHCs have a relatively high level of organizational resilience. CHC’s organizational resilience has a positive association with primary care nurses’ perception of psychological safety, organizational and professional commitment and organizational citizenship behaviours, but is not associated with primary care nurses’ depression and work exhaustion status. The primary care manager should build resilient organizations to enhance nurses’ psychological safety, commitment and behaviours.

## Supplementary Material

czae091_Supp

## Data Availability

The data underlying this article will be shared on reasonable request to the corresponding author.

## Abbreviations

CHCs = community health centres

PROMIS = The Patient-Reported Outcomes Measurement Information System

HLM = hierarchical linear regression model

VIF = variance inflation factor

ICC = intraclass correlation coefficient

## Reflexivity statement

Our research team maintains a balanced gender composition, comprising two male and four female researchers. Our team exhibits a diverse range of qualifications and expertise. R.M. has strong expertise in health care management in business school, W.W. has expertise in primary care policy in public policy school. The other four members are either research assistant or PhD students in the health policy program. This amalgamation of seniority and expertise enriches the depth and breadth of our research. Our team members originate from various countries, both China nd Australia, each contributing unique cultural perspectives to our research. Regardless of gender, nationality or seniority, all authors were actively engaged in defining the data collection tools, selecting the analysis strategy, interpreting the findings and contributing to the manuscript’s writing.
