# Supplementary material for: Organizational resilience and primary care nurses’ work conditions and well-being: a multilevel empirical study in China
Source: Health Policy Plan. 2024 Sep 16;39(10):1065–73. doi: 10.1093/heapol/czae091 (PMC11562115; doi:10.1093/heapol/czae091)
Supplement: czae091_Supp [file czae091_supp.zip › Supplementary files.docx]

**Supplementary File to**

**“****Organizational resilience and primary care nurses’ work conditions and wellbeing: a multilevel empirical study in China”**

**Entries for measured variables in the survey**

| **Variables** | **Items** |
| --- | --- |
| **Organizational resilience-Adaptive capacity** | Employees are regularly encouraged to move between departments/sections to enhance their work experience. |
|  | Employees have the right to prioritize access to knowledge and information on how to respond to emergencies. |
|  | Employees know how to solve problems creatively when faced with unexpected situations. |
|  | Employees have access to key information about how to respond to emergencies in a variety of ways. |
|  | Managers provide good leadership in the face of problems or challenges. |
|  | Even in the absence of consultation, employees will accept the decisions made by managers in response to a crisis. |
|  | Employees are encouraged to challenge themselves and improve themselves at work. |
|  | Employees are often rewarded for creative thinking and behavior. |
| **Organizational resilience-Planning capacity** | It is not enough to have contingency plans; they must be tested to ensure they are effective. |
|  | Employees are able to take time to train on how to respond to emergencies at work. |
|  | The organization is equipped with adequate resources to deal with emergencies. |
|  | The organization has adequate internal resources to cover day-to-day operational activities. |
|  | The organization has the capacity to deal with emergencies in addition to its daily medical activities. |
|  | The organization can readily deploy internal resources to deal with emergencies with less bureaucratic interference. |
|  | The organization can use their employees' networks to quickly access external resources to address unexpected situations. |
|  | The organization has reached a consensus with other organizations to provide resources to each other in case of emergencies. |
| **Psychological safety** | If a colleague makes a mistake at work, he or she will fall into disrepute. |
|  | Colleagues always ask difficult questions about each other in the workplace. |
|  | Colleagues sometimes exclude people who don't (think/behave) like them. |
|  | Working with colleagues, everyone feels free to say what they want to say and try to do what they want to do. |
|  | Working with colleagues, people can easily get help from others. |
|  | No colleague will deliberately oppose others in order to show his or her personal ability or superiority. |
|  | Working with colleagues, my talents and potential can be realized and recognized. |
| **Organizational commitment** | I have a deep affection for the people and things in this organization. |
|  | I am happy to work in this organization now and in the future. |
|  | I feel obliged to remain in my unit. |
|  | I think the problems of my organization should also be my problems. |
|  | I think this organization means a lot to me. |
|  | This organization gives me a strong sense of belonging. |
| **Professional commitment** | My career is very desirable and I would never give it up |
|  | If I had to choose again, I'd still be in my current career. |
| **Self-directed learning** | I actively participate in various types of training and endeavor to improve my business level and the quality of my work. |
|  | I make use of my spare time to enrich my medical knowledge and improve my practice. |
|  | I consciously and humbly learn from other highly skilled medical personnel. |
| **Depression** | I have felt worthless. |
|  | I have felt helpless. |
|  | I have felt frustrated. |
|  | I have felt hopeless. |
| **Burnout** | I have a feeling of dread when I think about the work I have to do. |
|  | I feel physically tired when I work. |
|  | I lack enthusiasm for my work. |
|  | I feel emotionally exhausted at work. |

**Descriptions of covariates**

The age was recorded as a continuous variable. The gender was reported as male or female. The marital status of nurses was classified as yes (married or cohabiting) and no (divorced or widowed). Education level was categorized as high school or below, and college or above. Tenured position was coded into yes or no, indicating whether nurses have a tenured position.

Ownership was defined as which entity managed the community health center (CHC), coded as public hospital and government. The percentage of health professionals was calculated using health professionals divided by all staff in CHC. The organizational size was defined as the number of total staff in the CHC, which was categorized as ≤35, 36-55, 56-100, and >100. The number of medical equipment was described as the number of valuable equipment over $1,600, was recorded as ≤4, 5-10, 11-30, and >30.
